# Editorial: Probing the Cardiac Arm of the Baroreflex and Complementary Branches

**DOI:** 10.3389/fnins.2019.01422

**Published:** 2020-01-10

**Authors:** Alberto Porta, Maja Elstad

**Affiliations:** ^1^Department of Biomedical Sciences for Health, University of Milan, Milan, Italy; ^2^Department of Cardiothoracic, Vascular Anesthesia and Intensive Care, IRCCS Policlinico San Donato, Milan, Italy; ^3^Division of Physiology, Institute of Basic Medical Sciences, University of Oslo, Oslo, Norway

**Keywords:** heart rate variability, arterial blood pressure, sympathetic neural activity, cardiovascular control, peripheral resistances, autonomic nervous system, baroreflex sensitivity

Baroreflex (BR) is one of the most important mechanisms in short-term regulation of arterial pressure (AP) (Robertson et al., 2012). The BR has a key role in limiting excessive AP rises via the activation of a vagal reflex (Robertson et al., 2012) producing several consequences on physiological variables. The most frequently evaluated consequence is the lengthening of heart period (HP) (Pickering et al., 1972). Baroreflex is crucial in bipedal animals like humans to prevent AP drops while standing via a sympathetic activation eliciting the HP shortening (Montano et al., 1994; Cooke et al., 1999; Marchi et al., 2016; De Maria et al., 2018) and the increase of burst rate of integrated postganglionic efferent sympathetic nerve activity directed to muscles (Sundlof and Wallin, 1978; Cooke et al., 1999; Furlan et al., 2000; Marchi et al., 2016). The BR engagement limits excessive AP variability in both humans and animals (Bertinieri et al., 1988; Parati et al., 1988; Frankel et al., 1993; Porta et al., 2000; Fazan et al., 2005).

The clinical evaluation of the BR control started with Smyth et al. (1969) who provided a practical, even though invasive, way to characterize BR via the estimate of the baroreflex sensitivity (BRS), namely the magnitude of HP changes observed in response to a pharmacologically induced unit variation of systolic AP (SAP). This interventional method is predictive for clinical outcomes (La Rovere et al., 1998) but, as it is inherently both non-physiological and invasive, researchers proposed noninvasive, non-interventional, and non-pharmacological surrogate techniques based on spontaneous fluctuations of HP and SAP with the aim at enlarging and favoring clinical applications (Laude et al., 2004). Both interventional and non-interventional techniques made the BR assessment popular but they contributed to form the common belief that BR is coincident with its cardiac arm operating to keep AP constant via HP adjustments. However, cardiac BR (cBR) is neither the unique arm of the BR nor the most important one, given that recent heart transplanted patients can stand up (Smith et al., 1989; Karemaker and Wesseling, 2008) and technologies for baroreflex failure target directly vasomotor sympathetic nerves (Hosokawa and Sunagawa, 2016). One of the consequences of the view identifying the BR with cBR is the tendency of interpreting modifications of the mean AP experienced during everyday life in spite of homeostatic characteristic of the BR as a result of its noisy nature (Karemaker and Wesseling, 2008). Conversely, the stochastic nature of the BR might be the simple consequence of its complex and composite nature: indeed, since the BR can target several physiological variables including heart rate, sympathetic activity, peripheral resistances, cardiac contractility, and stroke volume just to mention a few (Smyth et al., 1969; Sundlof and Wallin, 1978; Casadei et al., 1992; Kienbaum et al., 2001; Yasumasu et al., 2005; Vaschillo et al., 2012; Borgers et al., 2014; Barbic et al., 2015; Elstad et al., 2015; Hosokawa and Sunagawa, 2016; Reyes del Paso et al., 2017; Porta et al., 2018) and since the functioning of all these branches is weakly correlated as it appears from the weak correlation among BRSs (Rudas et al., 1999; O'Leary et al., 2003; Dutoit et al., 2010; Taylor et al., 2015; Marchi et al., 2016), it is not surprising to observe that mean AP does not always obey to the homeostatic principle. The composite nature of the BR is compatible with the observation that short-term fluctuations of HP are not intimately and always linked to those of SAP (Diaz and Taylor, 2006).

The aim of this Research Topic is, on the one hand, to stress the composite nature of the BR and the need of overcome a description solely based on the assessment of the cBR and, on the other hand, the possibility to provide a more complete, and faithful, description of the BR based on the use of a multivariate integrated approach exploiting simultaneous recordings of several physiological variables and state-of-the-art signal processing techniques applied to their spontaneous fluctuations. Among the most relevant challenges that need to be faced to make this approach successful we recall the inherent difficulty posed by the small amplitude of the spontaneous SAP fluctuations in assuring a BR description uncorrupted by confounding mechanisms operating in causal directions incompatible with a BR engagement (Porta et al., 2000, 2013; Diaz and Taylor, 2006).

In this Research Topic the complexity and composite nature of the BR and its assessment is illustrated by the diversity in the contributions. They stress the relevance of the simultaneous assessment of cardiac and sympathetic arms of the BR in healthy subjects (Barbic et al.) and patients (Brunetta et al.), the different characteristics of the BR arms likely to contribute to their weakly correlated behaviors (De Maria et al.), the importance of the clinical information that can be derived from BR markers estimated from spontaneous variability (Bari et al. and Solaro et al.), the chance of elucidating the brainstem nuclei functioning involved in the modulation of the activity of all BR branches (Gerlach et al.), the importance of modeling the dynamical interactions among variables via modeling approaches accounting for directionality (Chalacheva et al.) and feedforward influences (Parati et al.), the possibility given by advanced signal processing tools to provide a more insightful description of the complex behavior of the cBR arm (de Boer and Karemaker) and to limit the effects of confounding factors (Silva et al.), and the opportunity of exploiting smart technologies to broaden the range of applications of BR monitoring (Lázaro et al.). We hope this Research Topic contributes to understanding the complex nature of the BR and its assessment.

## Author Contributions

AP and ME conceived the contribution, drafted the manuscript, edited and revised the manuscript, and approved the final version of the manuscript.

### Conflict of Interest

The authors declare that the research was conducted in the absence of any commercial or financial relationships that could be construed as a potential conflict of interest.
